# Assessment of knowledge and facility compliance with the WHO SAM Guidelines among health professionals in the Volta Region of Ghana

**DOI:** 10.4314/gmj.v59i4.5

**Published:** 2025-12

**Authors:** Ruth Dormediame, Pearl Kudexa, Beth Offei-Awuku, Freda Intiful, Matilda Asante, Laurene Boateng

**Affiliations:** 1 Department of Dietetics, School of Biomedical and Health Sciences, University of Ghana, Legon, Ghana; 2 University of Cape Coast Hospital, University of Cape Coast (UCC), Cape Coast, Ghana

**Keywords:** Severe Acute Malnutrition, WHO, Training, Healthcare delivery, Ghana

## Abstract

**Objective:**

The purpose of this study was to assess health staff's knowledge and facility compliance with the World Health Organisation (WHO) treatment guidelines for Severe Acute Malnutrition (SAM) in selected hospitals in the **Volta Region** of Ghana.

**Design:**

This study employed a cross-sectional design.

**Setting:**

Three hospitals in the Volta Region of Ghana: Ho Municipal Hospital, Ho Teaching Hospital and Adidome Government Hospital.

**Participants:**

All health staff, including doctors, dietitians, nurses, and health assistants/health aides, involved in the treatment of SAM in the pediatric wards of the selected hospitals (70 in all).

**Main outcome measure:**

Health staff's training and knowledge of the WHO protocol for management of SAM, as well as their facility's compliance with the feeding aspect of the WHO protocol.

**Results:**

Majority of respondents (84%) had adequate knowledge of the WHO protocol, whilst only 37.1% graded their facilities as compliant with the feeding aspect of the WHO protocol. Less than half (41.4%) of participants received training on the WHO protocol for SAM management. There were significant associations between the training of participants on the WHO protocol and their knowledge of the WHO treatment guidelines, as well as the facility's compliance with the feeding aspect of the guidelines.

**Conclusion:**

Despite adequate knowledge of the WHO SAM protocol among health professionals, the majority graded their facilities as non-compliant, highlighting the need for targeted training and institutional support to improve adherence and enhance SAM treatment outcomes in Ghana.

**Funding:**

None declared

## Introduction

Malnutrition in children leads to morbidity, mortality, impaired intellectual development, economic productivity reduction, and increased adult disease risk. Globally in 2020, 45.4 million children under 5 years old were wasted. Out of the number of children affected by wasting, 13.6 million were severely acutely wasted.^1^ During the same time period, 12.1 million (6.0%) children in Africa suffered from wasting, and out of these numbers, 3 million (1.5%) were severely wasted.^1^

According to the World Health Organisation (WHO),^2^ the treatment of severe acute malnutrition (SAM) involves feeding malnourished children with specialised therapeutic foods such as Formula 75 (F-75), Formula 100 (F-100) and Plumpy Nut. In inpatient care, treatment begins with the stabilisation phase, during which F-75 (75 kcal/100 ml), a low-protein, milk-based formula, is used. This is followed by the nutritional rehabilitation phase, where F-100 (100 kcal/100 ml) or Plumpy Nut may be administered. F-100 is also a milk-based formula but provides higher energy and protein than F-75. Plumpy Nut is a ready-to-use therapeutic food (RUTF), which, unlike the water-based F-75 and F-100, do not require water for preparation. This reduces the likelihood that RUTF will be contaminated by harmful microorganisms.^2^

The WHO protocol for SAM management has been proven to reduce mortality among children with SAM. However, in low-resource settings, the mortality rate for SAM remains high.^3^ Key reasons for case fatality in the inpatient management of SAM include a combination of insufficiently trained staff, poor teamwork and inadequate compliance with WHO treatment guidelines.^3^

In a study by Muzigaba and colleagues,^4^ poor management of SAM cases was linked to the lack of continuity in training of rotating clinicians, sporadic shortages of therapeutic resources, inadequate staffing levels after normal working hours and some organisational and system-wide challenges beyond the immediate control of clinicians.^4^ They therefore suggested that a verificatory study be conducted to corroborate the findings from their study. The United Nations Children's Fund (UNICEF) recommends that the most effective way to build and maintain the quality of severe acute malnutrition management is to train health care staff with experienced trainers.^5^ According to WHO, if the inpatient treatment guidelines for SAM are followed, the mortality rate should be less than 10%.^6^ Despite reported compliance with these guidelines, health centres in sub-Saharan Africa have reported mortality rates of 10–40% among severely malnourished hospitalised children.^7^ In two referral hospitals in Ghana, the mortality rate for SAM was 17.7%.^8^

Furthermore, the 2022 Ghana Demographic and Health Survey reported that the Volta Region of Ghana had 1.2% wasting among children under 5 years old.^9^ Thus, this study aimed to assess health staff's knowledge and compliance of their facilities with the WHO treatment guideline for severe acute malnutrition in selected hospitals in the Volta Region of Ghana.

## Methods

### Study design and sample

The study employed a cross-sectional design. Three hospitals from the Volta region of Ghana, Ho Municipal Hospital, Ho Teaching Hospital and Adidome Government Hospital, were purposively selected, due to proximity and presence of a SAM management unit. Respondents were selected using a total enumeration sampling approach. All health staff (doctors, dietitians, nurses, and health assistants/health aides) involved in the treatment of SAM in the pediatric wards of the selected hospitals were invited to participate in the study. Altogether, seventy (70) health staff (comprising 18 from Ho Municipal Hospital, nine from Adidome Government Hospital and 43 from Ho Teaching Hospital) participated in the survey from July-August 2022. Pretesting of all data collection instruments was done at a hospital with similar characteristics as the study hospitals, among 10 health professionals involved with SAM management, to validate the data collection instruments.

### Data collection and management

Socio-demographic information, such as age, gender, and professional qualifications, was obtained from respondents using a structured, interviewer-administered questionnaire.

A knowledge test based on the WHO treatment guideline for SAM was developed by the research team based on a literature review, community management of acute malnutrition (CMAM) guidelines and the WHO ten steps for management of severe acute malnutrition.

The data collection tool underwent both content and construct validation to ensure its relevance and alignment with the study objectives. Content validity was established through expert review (conducted by two research dietitians in academia and one senior clinical dietitian), while construct validity was ensured by aligning each item with theoretical constructs and established protocols outlined in the WHO and CMAM guidelines. The test required participants to respond true or false to a set of 40 statements (grouped into 10 themes) regarding the treatment guideline for SAM (Appendix 1). The total score for the test was 40 marks, and this was converted to a percentage for each participant. Based on a similar work by Mogre et al,^10^ where a score of 60% and above was regarded as adequate knowledge of the WHO protocol for SAM management, we categorized scores within the range of 100% – 90% as very good knowledge; 89% - 60% as good knowledge; average knowledge as 59% - 50% and low knowledge as < 50%.

Health staff's assessment of their facilities' compliance with the feeding aspect of the WHO protocol for management of SAM was based on their responses to four questions on the structured questionnaire (Appendix 2–Questions 17-20). Facility compliance with the WHO treatment guideline for SAM was determined if respondents answered all four questions on feeding practices in their facility correctly. If any question was answered incorrectly, the respondent was considered to have graded their facility as non-compliant.

Healthcare staff perceptions of the effectiveness of therapeutic foods in promoting rapid recovery in malnourished children were assessed using a 5-point Likert scale. The scale ranged from 1 (No effect) to 5 (Excellent effect), with intermediate points being 2 (Poor effect), 3 (Good effect), and 4 (Very good effect). An open-ended question was used to collect information on the challenges faced by health staff in feeding children with SAM. To ensure accuracy and consistency, interviewers received prior training on survey administration and data recording. Responses were recorded on paper-based forms and subsequently entered into a secure database on a password-protected computer and accessible only to authorised research personnel. The dataset was de-identified prior to analysis to maintain participant confidentiality. Unique study identification numbers were assigned to each respondent, and no personally identifiable information was retained.

### Data analysis

The Statistical Package for the Social Sciences (SPSS version 22.0)^11^ was used for data analysis. Frequencies and proportions were calculated for health staff's scores on knowledge of the WHO treatment guideline for SAM, compliance with the feeding component of the WHO protocol, and their perceptions of the effectiveness of therapeutic foods in helping malnourished children recover quickly. Pearson's Chi-square test of independence was used to assess whether associations existed between training on the WHO protocol for SAM management, health staff's knowledge of the protocol, facility compliance with the protocol, and perceptions of the effectiveness of therapeutic foods. The open-ended responses, which addressed the challenges of feeding SAM children, were analysed using inductive thematic analysis.

Our aim was to have a deeper understanding of the challenges health professionals faced in their facilities regarding feeding children with SAM. Specifically, we followed Braun & Clarke's six-phase framework, which includes familiarisation with the data, generating initial codes, searching for themes, reviewing themes, and defining and naming themes.^12^

### Patient and public involvement

No patients or members of the public were involved in the study.

### Ethical considerations and approval

Ethical clearance was obtained from the Ethics and Protocol Review Committee of the School of Biomedical and Allied Health Sciences, University of Ghana (protocol identification number: CHS-Et/M.11 – P4.5/2021-2022). Also, approval was obtained from the Ethics and Protocol Review Committees of the study hospitals. Consent was obtained from all respondents. The study's aim, objectives, expected role, confidentiality, and voluntary participation were explained to all eligible participants.

## Results

### Sociodemographic information

A total of 70 health staff participated in the study. There were 44 females (62.9%) and 26 males (37.1%). More than half (55.7 %) of participants were within the age group of 26-35 years. The majority (62.9%) of study participants were nurses, and 25 (35.8%) had more than 1 year of experience in SAM management (Table 1).

**Table 1:** Background characteristics of study participants

Characteristics	Frequency (n = 70)	Percentage (%)
**Age (years)**		
**18-25**	21	30.0
**26-35**	39	55.7
**36-45**	10	14.3
**Sex**		
**Male**	26	37.1
**Female**	44	62.9
**Professional Qualification**		
**Doctor**	5	7.1
**Dietitian**	17	24.3
**Nurse**	44	62.9
**Health Assistant / Aid**	4	5.7
**SAM Management Experience**		
**Less than a month**	16	22.9
**One month to six months**	12	17.1
**Six months to one year**	17	24.3
**One to two years**	9	12.9
**Above 2 years**	16	22.9

### Health staff's training and knowledge of the WHO treatment guideline for SAM

**Figure 1:**
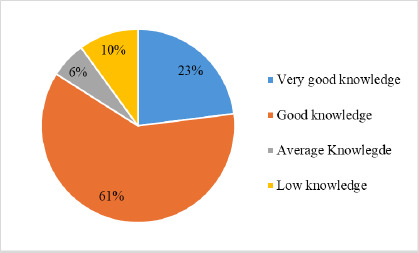
Knowledge level of study participants on the WHO protocol for SAM management

The majority (61%) of participants had good knowledge of the WHO protocol for management of SAM as shown in Figure 1. Ten per cent (10%) had low knowledge of the WHO protocol. Eighty-four per cent (84%) of them had adequate knowledge of the WHO treatment protocol. The average score of study participants in the assessment of their knowledge of the WHO protocol for SAM management is 75.8 ± 18.9. Based on the assessment, only 37.1% of participants were adhering to the feeding component of the WHO protocol for SAM management.

### Perception of health staff on the effectiveness of therapeutic foods

**Figure 2:**
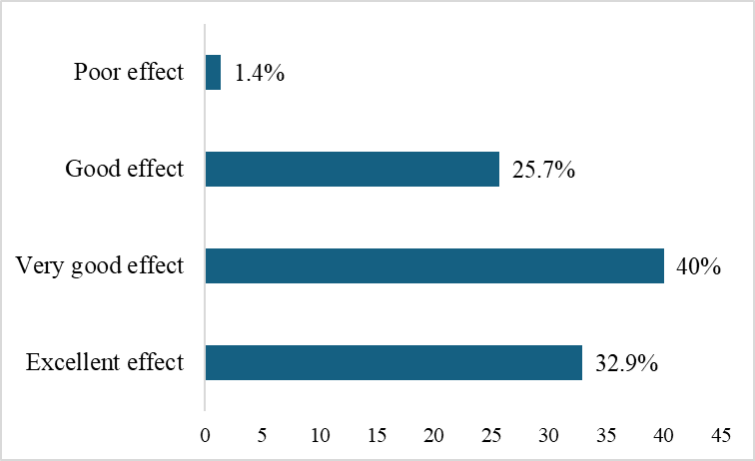
Perception of effectiveness of therapeutic foods by study participants

Forty per cent and 32.9% of study participants perceived therapeutic foods used in the management of SAM as having a very good effect and excellent effect, respectively, in helping SAM children recover quickly (Figure 2). Altogether, ninety-eight per cent (98.6%) of the participants perceived the therapeutic foods used in the management of SAM as an effective treatment. There was no response for ‘No effect.’

### Health staff training and its association with knowledge of the WHO protocol, facility compliance with the WHO protocol, and perceptions of the effectiveness of therapeutic foods

**Figure 3:**
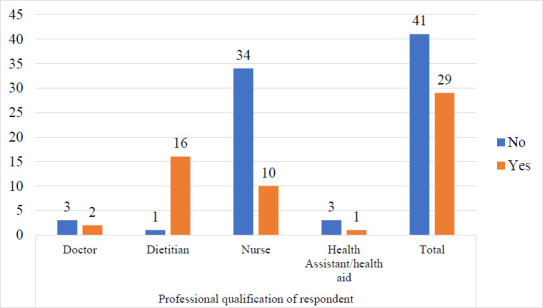
Health Staff's training on the WHO protocol for SAM management

Regarding training, the majority of dietitians (94.1%) had received training, compared with other health professions. The majority of dietitians were trained; however, overall, less than half (41.4%) of health staff had previously been trained in the WHO protocol, as shown in Figure 3.

There were significant associations between health staff training on the WHO protocol for SAM management and facility compliance with the feeding component of the protocol (P < 0.001) and with their knowledge of the protocol (P = 0.009). The association between training of health staff on the WHO protocol on SAM management and their perception of the effectiveness of therapeutic foods was, however, not significant (Table 2).

**Table 2:** Association between health staff's training and knowledge of WHO protocol, facility compliance with WHO protocol and perception of effectiveness of therapeutic foods

Characteristic	Trained (n=29)	Not trained (n=41)	P-value
**Professional Qualification**			
Doctor	2(6.9%)	3(7.3%)	P<0.001*
Dietitian	16(55.2%)	1(2.4%)	
Nurse	10(34.5%)	34(82.9)	
Health assistant/aid	1(3.4%)	3(7.3%)	
**Facility Compliance**			
Compliant	18(62.1%)	8(19.5%)	P<0.001*
Non-compliant	11(37.9%)	33(80.5%)	
**Perception of effectiveness of therapeutic foods**			
Excellent effect	13(44.8%)	10(24.4%)	P=0.101^b^
Very good effect	12(41.4%)	16(39.0%)	
Good effect	4(13.8%)	14(34.1%)	
Poor effect	0	1(2.4%)	
**Knowledge level**			
Very good knowledge	11(37.9%)	5(12.2%)	P=0.009^b^*
Good knowledge	16(55.2%)	27(65.9%)	
Average knowledge	2(6.9%)	2(4.9%)	
Low knowledge	0	7(17.1%)	

Significant at p ≤ 0.05: Pearson's Chi square

* p-value is significant

b Fisher's exact test

### Challenges faced by participants in feeding SAM children with therapeutic foods

**Figure 4:**
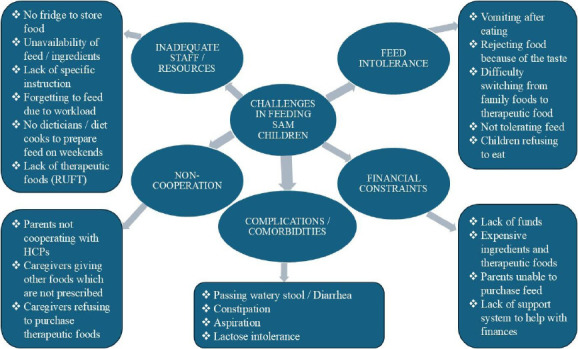
Challenges faced by participants in feeding SAM children with therapeutic foods in their facilities

Using an open-ended question format, healthcare professionals were asked to describe any challenges they encountered when feeding severely acutely malnourished children with therapeutic foods in their facility. Following inductive thematic analysis, five main themes emerged: (i) intolerance of therapeutic feeds by children, (ii) financial constraints, (iii) inadequate staff and resources, (iv) non-cooperation from parents/caregivers, and (v) complications and comorbidities. Figure 4 presents the various themes that emerged.

### Feed intolerance

One of the most common themes across professions was the intolerance of some of the children to therapeutic feeds. Most of the health professionals reported that some children do not like the taste of the food, hence they reject it, while others vomit after eating. One dietitian reported that:

*“Some children vomit right after eating*,” “*some children do not tolerate the new food since they are used to the taste of home foods.”*

### Inadequate staff and resources

Another theme reported particularly by the nurses was the inadequacy of staff and resources needed to help with feeding the SAM children. Some responses were as follows.

*“There are no diet cooks or dieticians to prepare feed on weekends*,” “*there are no storage facilities to preserve the feeds*,” “*feeds available sometimes are not adequate for the children”*.

### Financial constraints

Participants also mentioned the financial constraints faced by some parents and caregivers. Some participants reported that.

*“Parents are unable to afford ingredients for therapeutic foods”, “caregivers complain about how expensive the feed is”*.

### Complications and comorbidities

Another theme that emerged was complications such as diarrhoea and constipation that come with feeding the SAM children. Participants reported that some of these complications interfere with the feeding regimen and make feeding more difficult. Some reported that:

*“Diarrhoea in some of the children makes feeding difficult”, “constipation occurs 2 to 3 days after feeding is initiated”*.

### Non-cooperation

The fifth theme identified was the lack of cooperation between staff and parents or caregivers. Some participants noted that certain caregivers were hesitant or unwilling to adhere to the feeding plan, often due to perceived lack of improvement in the child's condition. Some specifically reported that:

*“Refusal of caregivers to come for follow-up”, “parents are reluctant to follow the feeding plan”, “caregivers refuse to cooperate with the Healthcare professionals”*.

These themes, some of which occurred simultaneously, reflect the challenges that healthcare professionals face in feeding children with SAM.

## Discussion

This study assessed health staff's knowledge and compliance with WHO guidelines for managing severe acute malnutrition (SAM) in three hospitals in Ghana. Female participants accounted for 62.9% of the study sample and were primarily nurses, reflecting their dominance in SAM management. Dietitians were the second-largest category of health professionals, accounting for 24.3% of participants. This reflects the inclusion of dietitians in the care of SAM in a region of Ghana outside the Greater Accra Region (GAR). A longstanding challenge for the dietetic profession in Ghana has been the concentration of dietitians in the GAR, leaving some regions without any dietitians.^13^ The majority of participants were aged 26-35 years, similar to a previous study by Ngoma in Malawi.^14^ The average knowledge score for the WHO protocol for severe acute malnutrition treatment was 75.8 ± 18.9, which is considered adequate knowledge according to similar categorisation by Mogre et al.^10^ The majority (84% of health staff) had adequate knowledge of the WHO treatment protocol, despite only 41.4% of them being formally trained on the protocol.

Our study also found that only 37.1% of participants graded their facilities as compliant with the WHO protocol for SAM management. This finding aligns with a similar study by Kenao et al.,^15^ which assessed the overall quality of SAM management, based on inputs, processes, and outcomes, and reported a quality score of 41.38%. The low facility compliance reported by health staff, despite adequate knowledge of the protocol, could have been better explained through direct observation of staff during the treatment of malnourished children, similar to methodologies employed in other studies.^14^,^15^ Additionally, engaging facility administrators and heads of SAM management units might have provided deeper insights into systemic or operational barriers affecting compliance.

Our findings revealed that nearly all participants (98.6%) perceived the therapeutic foods used to manage SAM as effective, although few adhered to the treatment protocol. The finding on their perception is, however, similar to a study by Tadesse et al.,^16^ who also found that caregivers and health workers perceived RUTF as an effective treatment for malnutrition.^16^

Regarding training, our study found that the majority of dietitians (94.1%) had received training on the WHO protocol for SAM management, in contrast to other health professions. This disparity highlights the uneven distribution of formal training across different professions. Additionally, the low levels of training among participants underscore the urgent need to expand formal training opportunities to enhance healthcare professionals' competence and confidence in managing SAM.^17^ In this study population, based on our findings, dietitians could be specially trained to serve as trainers for other healthcare professionals.

The open-ended enquiry on challenges faced by SAM children revealed several key issues. Children suffering from SAM often have compromised gut integrity and appetite issues, leading to refusal and difficulties in digesting therapeutic feeds.^18^ The findings of this study reinforce these concerns, as healthcare professionals reported frequent incidences of feed intolerance. Complications and underlying conditions may make it difficult for children to tolerate therapeutic foods. Children with SAM often present with multiple medical complications, including infections, electrolyte imbalances, and micronutrient deficiencies, making their nutritional rehabilitation complex. Healthcare professionals in this study highlighted the frequent occurrence of severe complications, which may complicate feeding.

The shortage of trained healthcare professionals and essential resources in managing SAM has been widely reported in the literature. Insufficient staffing and resources are a concern, as healthcare funds are often allocated to treatments rather than nutrition education programs or malnutrition prevention.^19^ According to a study by Kerac et al.,^20^ many healthcare facilities in low- and middle-income countries (LMICs) operate with limited staff, leading to high patient-to-provider ratios and compromised care. Findings from this study align with these concerns, as participants reported overwhelming workloads, insufficient personnel, and inadequate supplies of therapeutic foods and essential equipment. The lack of adequate training in SAM management exacerbates the situation, resulting in lower-quality care for affected children. A similar study in Bangladesh also found that overburdened staff, insufficient training, lack of functioning instruments and increasing patient load are major challenges in SAM management.^21^ To support proper recovery, governments and institutions should implement policies that allocate funds for adequate staffing, training and the provision of therapeutic foods for children with SAM.

Financial constraints significantly hinder the optimal care of children with SAM, particularly in low- and middle-income countries (LMICs). Previous research has high-lighted that both healthcare facilities and caregivers face economic burdens that limit access to specialised nutritional products and medical interventions.^22^ Findings from this study agreed with this, with healthcare workers citing the high costs of therapeutic foods and inadequate funding for essential services as barriers to effective treatment. Economic hardships among caregivers also limit their ability to provide adequate post-discharge nutrition, increasing the risk of relapse.

Non-cooperation between staff and parents or caregivers was another theme, which overlapped with financial constraints. Caregivers often lack awareness of the severity of SAM or adhere to traditional feeding beliefs that conflict with medical recommendations.^23^ In instances where manufactured therapeutic foods are in short supply, care-givers of children with SAM are often required to purchase ingredients for locally prepared alternatives. However, families unable to afford these ingredients may deviate from recommended treatment protocols, including secretly providing non-prescribed foods, which can compromise the effectiveness of nutritional rehabilitation. This study found that healthcare professionals frequently encountered resistance from caregivers, making it difficult to implement nutritional interventions effectively. Misconceptions about therapeutic feeding, cultural dietary preferences, superstitious beliefs and fear of medical treatments can sometimes contribute to non-compliance. Addressing these challenges requires education and community engagement targeted at caregivers of children with SAM, to explain the reasons behind certain instructions and improve adherence to treatment plans.

A major limitation of this study was the interview-based approach used to evaluate inpatient management of SAM, which may have introduced response bias. Also, compliance with the feeding aspect of the WHO protocol for SAM management was assessed based on respondents' answers to four structured questions. These responses reflect health staff's report of their facility's adherence to the protocol, rather than direct observation or audit of clinical practice.

Furthermore, the three hospitals where this study was conducted are all located in one of Ghana's 16 regions, which may limit the generalizability of the findings to other regions with different healthcare infrastructures and patient demographics.

The findings of this study highlight critical gaps in training, resource availability, and caregiver engagement in the management of severe acute malnutrition (SAM). For practice, our findings emphasise the need for regular, structured in-service training for all health professionals, especially leveraging dietitians as trainers. For healthcare policy, it calls for increased investment in nutrition services, including therapeutic food supplies and staffing, integration of SAM management into health curricula, and community education programs to improve caregiver cooperation.

## Conclusion

The study found a significant association (P<0.001) between healthcare staff training on the WHO protocol for managing severe acute malnutrition (SAM) and facility compliance with the feeding component of the protocol. Our findings highlight gaps in the implementation of WHO treatment guidelines for the management of SAM. We recommend that hospital policies should include mandatory regular in-service training on the WHO protocol for the management of SAM for all categories of health staff who work to manage SAM to ensure better compliance and better treatment outcomes.

## Appendix 1

### TEST - GENERAL KNOWLEDGE OF WHO PROTOCOL FOR THE MANAGEMENT OF SEVERE ACUTE MALNUTRITION

#### Please indicate whether the statements a – d are *true* or *false*

1. To treat/prevent hypoglycaemia
  a) Start treating immediately (true/false)
  b) Wait for caregiver's permission to treat hypoglycaemia (true/false)
  c) Treat hypoglycaemia with dextrose immediately (true/false)
  d) Feed with therapeutic foods at 2-4 hours intervals day and night (true/false)
2. To treat/prevent hypothermia
  a) Actively re-warm the child (true/false)
  b) Keep malnourished children warm during the day only (true/false)
  c) Keep malnourished children warm during the night only (true/false)
  d) Keep malnourished children warm day and night (true/false)
3. To treat/prevent dehydration
  a) Do not give IV fluids except in shock (true/false)
  b) Rehydrate more slowly than usual (true/false)
  c) Rehydrate with ReSoMal (true/false)
  d) Too much fluid can kill (true/false)
4. To correct electrolyte imbalance
  a) Give extra potassium daily (true/false)
  b) Give extra magnesium daily (true/false)
  c) When rehydrating, give low sodium rehydration fluid (ReSoMal) (true/false)
  d) When rehydrating, give high sodium rehydration fluid (true/false)
5. To treat/prevent infection
  a) Give antibiotics routinely to all severely malnourished children (true/false)
  b) Don't give antibiotics routinely to all severely malnourished children (true/false)
  c) Antibiotics are given to treat hidden infections and prevent death (true/false)
  d) Adhere to strict hygienic practices (true/false)
6. To correct micronutrient deficiencies
  a) Give extra vitamin A, zinc, copper, folic acid and multivitamins (true/false)
  b) Don't give extra vitamin A, zinc, copper, folic acid and multivitamins (true/false)
  c) Give iron before the child is in the rehabilitation phase (true/false)
  d) Do not give iron until the child is in the rehabilitation phase (true/false)
7. To start cautious feeding
  a) Give small amounts of F75 (true/false)
  b) Give F75 every 2 or 3 hours during the day and night (true/false)
  c) Giving F75 will help resolve bilateral pitting oedema if it is present (true/false)
  d) Giving F75 does not help resolve bilateral pitting oedema if it is present (true/false)
8. To achieve catch-up growth
  a) Give as much F100 or ready-to-use therapeutic food (RUTF) as the child wants (true/false)
  b) Give as much F75 as the child can eat (true/false)
  c) Give F100 six (6) or eight (8) times a day (true/false)
  d) Give recommended amount of RUTF (true/false)
9. To provide sensory stimulation and emotional support
  a) Provide loving care (true/false)
  b) Provide play and stimulation (true/false)
  c) Leave the child to be by himself/herself most of the time (true/false)
  d) Sensory stimulation and emotional support will improve mental development (true/false)
10. To prepare for follow-up after recovery
  a) Teach mothers what to feed at home (true/false)
  b) Take the contact number and accurate address of mother/caregiver (true/false)
  c) Let mothers know you will make follow ups (true/false)
  d) Do not let mothers know you will make follow ups (true/false)

## Appendix 2

### QUESTIONNAIRE - SOCIODEMOGRAPHIC INFORMATION & COMPLIANCE WITH WHO PROTOCOL IN ADMINISTERING THERAPEUTIC FOODS

1. Name of hospital.
  ………………………………………………………………………………………………..
2. Region in which the hospital is
  [ ] Volta region
3. Age of respondent
  [ ] 18-25 years
  [ ] > 25-35 years
  [ ] > 35-45 years
  [ ] > 45 years
4. Sex of respondent
  [ ] Male [ ] Female
5. Professional qualification of respondent
  [ ] Doctor
  [ ] Dietitian
  [ ] Nutrition officer
  [ ] Nurse (Unit Manager/in-charge)
  [ ] Nurse (general)
  [ ] Health assistant
  [ ] Health aid
  [ ] Other (Please specify) …………………………………….
6. Rank of respondent (please mention your rank)
  ………………………………………………………………………………………………
7. On average, how many severe acute malnutrition cases does your hospital manage annually? (for dieticians/nutrition officers only) ……………………………………….
8. Out of the average number of severe acute malnutrition cases managed annually how many recovered? (for dieticians/nutrition officers only) ……………………….
9. Out of the average number of severe acute malnutrition cases managed annually how many died? (for dieticians/nutrition officers only) ………………………..
10. How long have you been involved in the management of severely acutely malnourished children?
  [ ] Less than a month
  [ ] One month to six months
  [ ] Between six months and a year
  [ ] Between one year and two years
  [ ] Above two years
11. Do you know the WHO protocol for the management of severe acute malnutrition?
  [ ] Yes [ ] No [ ] Not sure
12. Do you know the ten (10) steps in the management of severe acute malnutrition?
  [ ] Yes [ ] No [ ] Not sure
13. In your opinion is the WHO protocol for the management of severe acute malnutrition being followed in your facility?
  [ ] Yes [ ] No [ ] Don't know
14. Are you trained (externally or internally) on the WHO protocol for management of severe acute malnutrition?
  [ ] Yes [ ] No
15. Do you know the therapeutic foods that are used in the management of severe acute malnutrition?
  [ ] Yes [ ] No
16. If Yes (to question no. 15) please name the therapeutic foods.
  a) ……………………………………………….
  b) ……………………………………………….
  c) ……………………………………………….
  d) ……………………………………………….
  e) ……………………………………………….
17. In your hospital, at what interval is F75 fed to malnourished children?
  [ ] 2 or 3 hourly [ ] 4 or 5 hourly [ ] 5 hourly
18. In your hospital at what interval is F100 fed to malnourished children?
  [ ] 2 hourly [ ] 3 or 4 hourly [ ] 5 hourly
19. In your hospital at which stage of treatment is RUTF given to severely acutely malnourished children?
  [ ] Stabilization stage [ ] Rehabilitation stage
20. In your hospital at which stage of treatment is F75 usually given to severely acutely malnourished children?
  [ ] Stabilization stage [ ] Rehabilitation stage
21. Who prepares the therapeutic foods (mixing other ingredients with hot water) before it is fed to the sick child? (multiple choices possible)
  [ ] Dietitian
  [ ] Nutrition officer
  [ ] Nurse (Unit Manager/in-charge)
  [ ] Nurse (general)
  [ ] Health assistant
  [ ] Health aid
  [ ] Other (Please specify) ………………….……………….
22. Which of these people specifically feeds the severely acutely malnourished child on admission at your facility (multiple choices possible)?
  [ ] Dietitian
  [ ] Nutrition officer
  [ ] Nurse (Unit Manager/in-charge)
  [ ] Nurse (General)
  [ ] Health assistant
  [ ] health aid
  [ ] Child's mother/caregiver
  [ ] Other (Please specify) ………………….……………….
23. Are there any challenges you face in feeding severely acutely malnourished children with therapeutic foods in your facility?
  [ ] Yes [ ] No
24. If Yes (to question no. 23) please briefly outline the challenges
  …………………………………………………………………………………………………………………………
  …………………………………………………………………………………………………………………………
  …………………………………………………………………………………………………………………………
  …………………………………………………………………………………………………………………………
  …………………………………………………………………………………………………………………………
  …………………………………………………………………………………………………………………………
  …………………………………………………………………………………………………………………………
  …………………………………………………………………………………………………………………………
  …………………………………………………………………………………………………………………………

#### 5-POINT LIKERT SCALE FOR “PERCEPTION OF HEALTH STAFF ON THE EFFECTIVENESS OF THERAPEUTIC FOODS IN HELPING MALNOURISHED CHILDREN RECOVER QUICKLY”

On a scale of 1 to 5 how would you rate the effectiveness of therapeutic foods in helping malnourished children recover quickly?

[ ] 1
[ ] 2
[ ] 3
[ ] 4
[ ] 5

*Where 1 = No effect; 2 = Poor effect; 3 = Good effect; 4= Very good effect and 5= Excellent effect
